# A Novel Bearing Fault Diagnosis Method Based on Few-Shot Transfer Learning across Different Datasets

**DOI:** 10.3390/e24091295

**Published:** 2022-09-14

**Authors:** Yizong Zhang, Shaobo Li, Ansi Zhang, Chuanjiang Li, Ling Qiu

**Affiliations:** 1 School of Mechanical Engineering, Guizhou University, Guiyang 550025, China; 2 State Key Laboratory of Public Big Data, Guizhou University, Guiyang 550025, China; 3 School of Computer Science and Technology, Guizhou University, Guiyang 550025, China

**Keywords:** fault diagnosis, few-shot, transfer learning, across different datasets

## Abstract

At present, the success of most intelligent fault diagnosis methods is heavily dependent on large datasets of artificial simulation faults (ASF), which have not been widely used in practice because it is often costly to obtain a large number of samples in reality. Fortunately, various faults can be easily simulated in the laboratory, and these simulated faults contain a lot of fault diagnosis knowledge. In this study, based on a Siamese network framework, we propose a bearing fault diagnosis based on few-shot transfer learning across different datasets (cross-machine), using the knowledge of ASF to diagnose bearings with natural faults (NF). First of all, the model obtains a good feature encoder in the source domain, then defines a fault support set for comparison, and finally adjusts the support set with a very small number of target domain samples to improve the fault diagnosis performance of the model. We carried out experimental verification from many aspects on the ASF and NF datasets provided by Case Western Reserve University (CWRU) and Paderborn University (PU). The results show that the proposed method can fully learn diagnostic knowledge in different ASF datasets and sample numbers, and effectively use this knowledge to accurately identify the health state of the NF bearing, which has strong generalization and robustness. Our method does not need second training, which may be more convenient in some practical applications. Finally, we also discuss the possible limitations of this method.

## 1. Introduction

Bearings are indispensable parts of much important machinery and equipment, which may lead to serious economic losses and casualties in the event of failure [1]. Therefore, it is essential to obtain the state of the bearing quickly and accurately. In recent years, machine learning has been applied to intelligent fault diagnosis of bearings because of its powerful ability. Nowadays, many well-known machine learning methods, such as support vector machine (SVM) [2], deep Boltzmann machine (DBM) [3], convolution neural network (CNN) [4], generate adversarial network (GAN) [5], and so on, have achieved excellent results. The success of most studies, however, are heavily dependent on a large number of artificial simulated faults (ASF) data, which has the following two conditions: (1) there is a large amount of marked data with fault information; and (2) the training data and testing data come from the same probability distribution. However, for a variety of reasons [6], it is impractical to obtain a large number of actual fault data in the real world, which cannot meet the first condition; the second condition cannot be satisfied because of the great difference in the feature distribution between the ASF and natural faults (NF). Therefore, many research results are not applicable to the working environment of real machines and cannot be widely applied in industrial production.

Recently, some researchers have tried to expand the amount of data by means of data over-sampling [7,8] and data generation [9] to solve the dilemma of limited fault data. However, these methods focus on the size of fault data and the data quality cannot be guaranteed, so their contribution to improving the current intelligent fault diagnosis performance is limited. For instance, due to the marginalization of distribution, there is a strong linear relationship between the generated data samples and training samples [10]. Subsequently, some scholars focused on few-shot learning (such as Matching networks [11], Siamese networks [12] and Relation networks [13]) and transfer learning. Few-shot learning is expected to have the same ability as human beings in the process of recognizing new species, acquiring knowledge based on a few instances and guiding new tasks [14]. The diagnostic knowledge that transfer learning can learn in the source domain is used in the target domain [15]. Few-shot learning and transfer learning are considered to be the most promising fault diagnosis technologies for practical applications, and they have been the main research hotspots in recent years [16].

In the aspect of few-shot learning, Ren et al. [17] proposed a capsule automatic encoder model based on automatic encoder and capsule network. Experiments show that the model has the ability to extract a variety of important features from a small number of samples, and can identify fault categories quickly and accurately. Zhang et al. [18] proposed a Siamese network model with a first-layer wide kernel convolution network. Through experimental verification under the conditions of limited data, new fault categories and noise, good fault identification accuracy is achieved. Li et al. [19] used sparse automatic encoders based on deep non-negative constraints to perform diagnosis under the condition of a small amount of fault data, and achieved certain results, but the classification accuracy is significantly reduced in the case of very few samples. Feng et al. [20] proposed a semi-supervised attention-attracting meta-learning network, which uses unlabeled data to refine the model and accurately identify faults. Li et al. [21] proposed a new model-agnostic meta-learning method for fault diagnosis under complex working conditions, and acquired knowledge through the diagnosis task of known working conditions to quickly diagnose bearing faults under unknown operating conditions. Yu et al. [22] integrated the few-shot learning strategy into a multi-label convolutional neural network for bearing fault diagnosis, and completed the diagnosis task with limited samples. In addition, many scholars [23,24,25,26] have proposed different few-shot learning methods to achieve fault diagnosis, but most of these methods rely on appropriate laboratory artificial fault data.

In the aspect of transfer learning, Wang et al. [27] developed a novel transfer learning method based on a Siamese network, and used the label trimming method to improve the classification performance of the model under different working conditions and cross bearing positions. He et al. [28] designed a new type of deep multi-wavelet automatic encoder to extract the knowledge of the source domain which is similar to the feature distribution of the target domain for fault diagnosis in the new domain. Liu et al. [29] proposed a new adversarial network focusing on the performance of bearing fault diagnosis under different working conditions, and enhanced the domain adaptability through the conditional countermeasure mechanism to improve the diagnosis effect. In addition, some scholars have tried to transfer from ASF to NF. For example, Wu et al. [30] used six different fine-tuning-based methods and a meta-learning model to carry out artificial-natural fault transfer experiments. The results are compared, and it is concluded that meta-learning is better in the relatively simple finite sample transfer task. Wang et al. [6] proposed an artificial-natural fault transfer learning method based on the reinforcement relation network (RRN), and improved the classification performance of the network by label smoothing and AdaBound algorithm. The data used in the above two articles, however, are from the same machine. As a result, some researchers have tried to transfer diagnostic knowledge from one machine to another. Yang et al. [31] proposed a feature-based transfer neural network to reduce the distribution differences and inter-class distances of the learned transferable features through multi-layer domain adaptation and regularization conditions for pseudo-label learning, utilizing the diagnostic knowledge of laboratory machines to identify the health status of real-case machines. Liang et al. [32] proposed a depth domain adaptive transfer learning network and carried out experiments on the fault data of different machines, it is proved that it is effective to collect tagged fault data from one machine for training and to test another machine.

To sum up, although few-shot learning has achieved certain achievements in fault diagnosis with limited samples, these achievements are based on the standard dataset of ASF, and there is a great difference between real fault and simulated fault, so it cannot be directly applied to real industrial machines. In the current research on transfer learning, whether between different working conditions or between ASF and NF, the source domain and target domain of most experiments come from the same dataset (the source and target domain data is collected on the same test bench or machine) and follow the same feature distribution. This ignores a major problem: if you want to apply it to a real machine, you need to obtain a large amount of appropriate source domain data in the same real machine, which is not feasible. Therefore, some researchers try to obtain a large amount of fault data from a machine that is convenient for data collection, and extract diagnostic knowledge from it to identify the health status of another machine, and we believe that this is a feasible method to solve the problem that a large number of fault data cannot be obtained in real industrial production. The reason for this is that it is relatively easy to obtain ASF data in the laboratory, which includes the diagnostic knowledge of real machine bearings.

In this paper, we propose a bearing fault diagnosis method based on few-shot transfer learning across different datasets (cross-machine) inspired by the fine-tuning-based method. Our model is based on the framework of a Siamese network and has the ability of few-shot learning. First of all, the model is trained with the ASF data, and the available diagnosis knowledge is learned. Then, a fault support set for comparison is defined and it is assumed that a very small number of NF samples can be obtained. These few NF samples are input directly into the support set or replace the original samples to improve the generalization ability of the model. Finally, the knowledge of ASF is used to effectively identify the health state of the new machine bearings. The main innovations and contributions of this paper are as follows:

(1) In view of the problems that most of the current intelligent fault diagnosis methods cannot be directly applied to industry, a few-shot transfer learning method across different datasets is proposed, which can use the diagnostic knowledge learned from ASF data to effectively identify the health state of the new machine bearings.
(2) For the first time, a very small number of target domain samples are used to replace the original samples of the support set in fault diagnosis, which improves the generalization ability of the model, and has very high stability and accuracy even in different datasets (ASF-NF) with great differences in feature space distribution.
(3) Several experiments are designed to compare and verify many aspects of the proposed method, which has achieved the expected results, and our method does not need secondary training, which will be more convenient.

The structure of this paper is as follows: Section 2 introduces the theoretical background of the method proposed in this paper. Section 3 introduces the proposed method and our model. Section 4 carries on the experiment and analysis from different aspects. Section 5 gives the main conclusions.

## 2. Basic Theory

### 2.1. Few-Shot Learning Strategy

**Figure 1:**
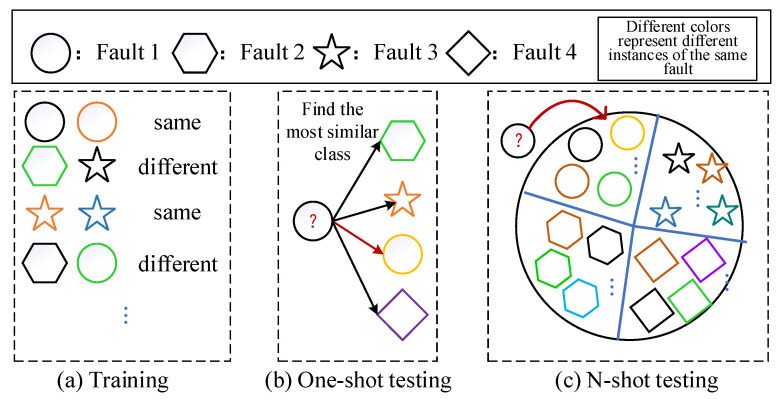
General strategies of few-shot learning.

When human beings recognize a new thing, they may only need to learn knowledge from a few instances to be able to accurately identify such things. Few-shot learning is proposed in order to acquire this human skill. The general strategy of few-shot learning based on a Siamese network is shown in Figure 1. Different from the general deep learning strategy, the input during training is a pair of the same or different samples $\left(x_{1},x_{2}\right)$, one only needs to label the sample pairs $\left(x_{1},x_{2}\right)$ with the same or different class. The output is the probability of similarity between sample pairs $\left(x_{1},x_{2}\right)$. When testing, there are mainly two strategies: one-shot k-way and N-shot k-way. One-shot k-way refers to the *k* categories in the support set, each class has only one instance; and N-shot k-way means that there are *k* categories in the support set, and each class has *N* instances.

In the one-shot k-way test, a test sample $\hat{x}$ that need to be classified and a support set are given, the support set is defined as shown in Equation (1). Next, the model judges the similarity between samples $\left(x_{1},x_{2},x_{3},\ldots ,x_{k}\right)$ in the support set and the test sample $\hat{x}$, and selects the highest similarity as the same class of $\hat{x}$, as shown in Equation (2).

(1) $$S=\left\{\left(x_{1},y_{1}\right),\ldots ,\left(x_{k},y_{k}\right)\right\}$$

The *y* is the label of the class, *k* represents the *k*th fault class.

(2) $$C\left(\hat{x},S\right)=\mathrm{argmax}\left(P\left(\hat{x},x_{c}\right)\right),x_{c}\in S,$$

The P is the probability of similarity, C is the fault class most similar to the test sample $\hat{x}$.

In the N-shot k-way test, there are *k* classes in the support set, each class has *N* different instances, such as shown in Equation (3), and the support set is shown in Equation (4).

(3) $$\left\{\begin{array}{c} \begin{array}{c} H_{1}=\left\{\left(x_{1}^{1},y_{1}^{1}\right),\left(x_{1}^{2},y_{1}^{2}\right)\ldots ,\left(x_{1}^{N},y_{1}^{N}\right)\right\}\\ H_{2}=\left\{\left(x_{2}^{1},y_{2}^{1}\right),\left(x_{2}^{2},y_{2}^{2}\right)\ldots ,\left(x_{2}^{N},y_{2}^{N}\right)\right\}\\ \ldots \ldots \ldots \ldots \ldots \ldots \ldots \ldots \ldots \ldots \ldots \ldots \ldots \ldots \ldots \ldots \ldots \end{array}\\ H_{k}=\left\{\left(x_{k}^{1},y_{k}^{1}\right),\left(x_{k}^{2},y_{k}^{2}\right)\ldots ,\left(x_{k}^{N},y_{k}^{N}\right)\right\} \end{array}\right.$$

The *H* is a set containing multiple instances of the same class, *k* represents the *k*th fault class, *N* represents the *N*th instance in the same fault class.

(4) $$S_{k}=\left\{H_{1},H_{2}\ldots ,H_{k}\right\}$$

The model will judge the similarity between the k*N instances of the support set and the test samples $\hat{x}$, and select the highest similarity as the same class of $\hat{x}$, as shown in Equation (5).

(5) $$C\left(\hat{x},S_{k}\right)=\mathrm{argmax}\left(P\left(\hat{x},x_{c}\right)\right),x_{c}\in S_{k}$$

Here *P* and *C* are the same as Equation (2), but the difference lies in the difference between *S* and $S_{k}$.

### 2.2. Fine-Tuning-Based Method

The main goal of fault diagnosis based on transfer learning is to transfer the learned knowledge from the source domain to the target domain. Among the many current transfer learning strategies, the fine-tuning-based method has been widely studied and proved to be effective. We are inspired by the fine-tuning-based method and put forward our method strategy.

**Figure 2:**
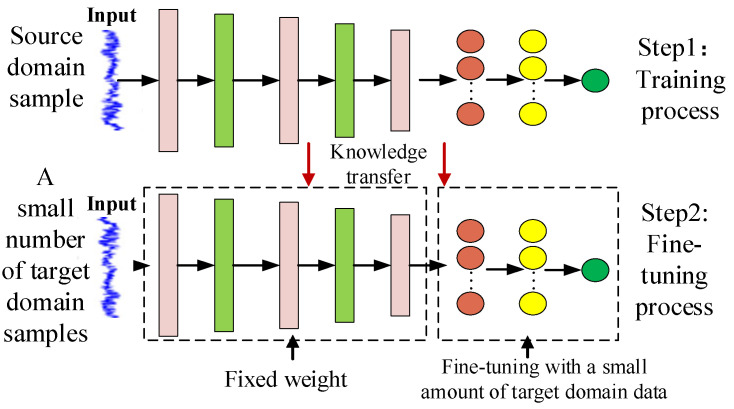
Transfer learning based on fine-tuning.

The learning process of fine-tuning-based method is divided into two stages. First, the network model learns the knowledge of diagnosis in the source domain; then, fine-tuning the full connection layer in the target domain to obtain a new classifier as shown in Figure 2.

## 3. The Proposed Method

### 3.1. The Proposed Few-Shot Transfer Learning Methods

**Figure 3:**
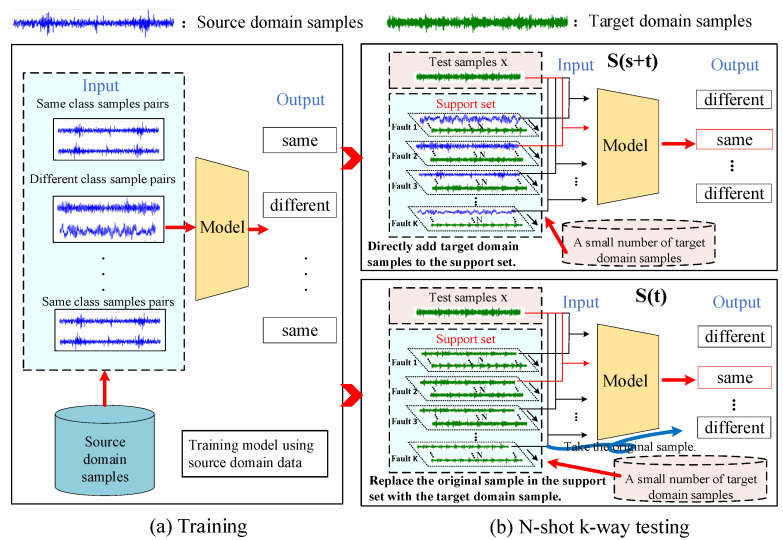
The proposed few-shot transfer learning methods.

We are inspired by the fine-tuning-based method and put forward our method strategy. From Section 2, we can see that the support set plays an important reference role in the Siamese network. The test sample x examples are always compared with the samples in the support set, and the most similar examples in the support set are selected for classification, as shown in Equations (2) and (5). In the few-shot transfer learning based on a Siamese network, we assume that a small amount of target domain data has been obtained and use them to adjust the support set, as in Figure 3. The following two few-shot transfer learning methods are proposed.

(1) S(s+t): Directly add target domain samples to the support set.

This method adds a very small amount of target domain samples $\left(x_{t},y_{t}\right)$ to the original support set after the training with source domain data, and finally tested. In this case, the expression of the support set is Equation (6). In this paper, we uniformly use S(s+t) to denote the method of directly add target domain samples to the support set.

(6) $$S=\left\{\left(x_{s1},y_{s1}\right)..\left(x_{sk},y_{sk}\right),\left(x_{t1},y_{t1}\right)..\left(x_{tk},y_{tk}\right)\right\}$$

The *s* in $\;x_{s}$ represents from the source domain, *t* in $\;x_{t}$ represents from the target domain.

(2) S(t): Replace the original sample in the support set with the target domain sample.

In this method, after training the model with source domain data, a very small number of target domain samples are used to replace the original samples in the support set, and the model is finally tested. At this point, the support set is shown in Equation (7). In this paper, we uniformly use S(t) to denote the replacement of the original sample in the support set with the target domain sample.

(7) $$S=\left\{\left(x_{t1},y_{t1}\right),\left(x_{t2},y_{t2}\right)\ldots ,\left(x_{tk},y_{tk}\right)\right\}$$

The *t* in $\;x_{t}$ represents the target domain.

### 3.2. Model

**Figure 4:**
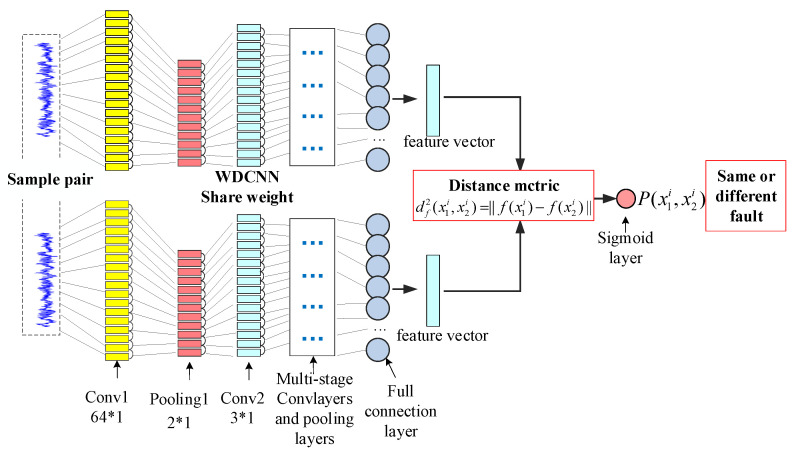
Few-shot learning model based on a Siamese network.

Figure 4 shows the model we use. This is a Siamese network with a deep convolution neural network with a wide first layer core (WDCNN). In this model, the two WDCNN have the same structure and parameters, and the weights are shared. The setting of the WDCNN network architecture is shown in Table 1, which is consistent with the setting in reference [33]. This design strategy is used because the vibration signal is more sensitive to the overall correlation in the time domain or frequency domain, and the useful information in the signal will be lost if the first layer core is too small, and because all layers are small cores which may be affected by high-frequency noise common in the industrial environment, resulting in poor performance of feature coding. It is proved that WDCNN with the first layer of wide kernel has good anti-noise ability, generalization ability and robustness. The model consists of a series of convolution layers, the step size of the first layer is set to 16, and the step size of the other layers is fixed to 1. In order to optimize the performance of the model, the number of convolution filters is a multiple of 16. In the previous convolution layer, the Relu activation function is used to encode the features, and the full connection layer uses the sigmoid activation function to map the features.

Input is a pair of samples $\left(x_{1},x_{2}\right)$, which can be the same or different. The output is the probability of similarity between the sample pairs. Firstly, the metric distance between the outputs of the network is optimized by Equation (8), where f represents a deep convolution network. Equation (9) determines the probability of similarity, where sigm represents the Sigmoid function and FC is a dense fully connected layer.

(8) $$d_{f}^{2}\left(x_{1}^{i},x_{2}^{i}\right)=\|f\left(x_{1}^{i}\right)-f\left(x_{2}^{i}\right)\|$$

(9) $$P\left(x_{1}^{i},x_{2}^{i}\right)=\mathrm{sigm}(\mathrm{FC}\left(d_{f}^{2}\left(x_{1}^{i},x_{2}^{i}\right)\right)$$

Let *M* represents the minibatch size, where *i* indexes the *i*th minibatch, let $y\left(i\right)=\left(x_{1}^{\left(i\right)},x_{2}^{\left(i\right)}\right)$ be a length-M vector which contains the labels for the minibatch. Now we assume $y\left(i\right)$ equal to 1 when $\left(x_{1}^{\left(i\right)},x_{2}^{\left(i\right)}\right)$ is the same class, and $y\left(i\right)$ equal to 0 when $\left(x_{1}^{\left(i\right)},x_{2}^{\left(i\right)}\right)$ is different class. We impose a regularized cross-entropy objective on our binary classifier of the following form:

(10) $$\begin{array}{c} L\left(x_{1}^{\left(i\right)},x_{2}^{\left(i\right)}\right)=y\left(i\right)\log\left(p\left(x_{1}^{\left(i\right)},x_{2}^{\left(i\right)}\right)\right)+\\ \begin{array}{c} \left(1-y\left(i\right)\right)\log\left(1-p\left(x_{1}^{\left(i\right)},x_{2}^{\left(i\right)}\right)\right)\\ +\lambda^{T}|w{|}^{2}\; \end{array} \end{array}$$

The optimizer we chose is Adam, which calculates individual adaptive learning rates. Update parameters through Equation (11):

(11) $$\begin{array}{c} m_{w}^{\left(T+1\right)}|=\beta_{1}m_{w}^{\left(T\right)}+\left(1-\beta_{1}\right)\nabla_{w}L^{\left(T\right)}\\ v_{w}^{\left(T+1\right)}|=\beta_{2}v_{w}^{\left(T\right)}+\left(1-\beta_{2}\right){\left(\nabla_{w}L^{\left(T\right)}\right)}^{2}\\ {\hat{m}}_{w}|=\frac{m_{w}^{\left(T+1\right)}}{1-{\left(\beta_{1}\right)}^{T+1}}\\ {\hat{v}}_{w}|=\frac{v_{w}^{\left(T+1\right)}}{1-{\left(\beta_{2}\right)}^{T+1}}\\ w^{\left(T+1\right)}|=w^{\left(T\right)}-\eta \frac{{\hat{m}}_{w}}{\sqrt{{\hat{v}}_{w}}+\epsilon } \end{array}$$

where $w^{\left(T+1\right)}$ means the parameters at epoch *T*, $L^{\left(T\right)}$ is the loss function, $\beta_{i}$ is the forgetting factor of the *i*th moment of the gradient, m and v are moving averages.

## 4. Experiment and Results

### 4.1. Data Introduction and Processing

**Figure 5:**
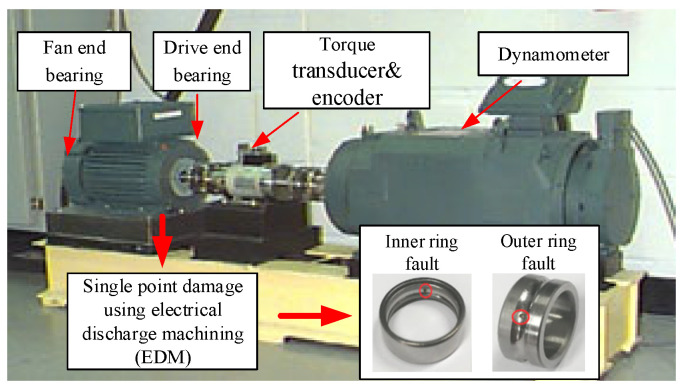
Bearing test bench of CWRU.

Like most deep learning algorithms, in order to confirm our proposed transfer learning strategy, we need to prepare appropriate data samples. We selected the data provided by Case Western Reserve University (CWRU) [34] as the ASF datasets, that is, the source domain. They are collected from the experimental platform of CWRU (shown in Figure 5), and all use the single point damage of electro-discharge machining (EDM). The vibration acceleration signal of the faulty bearing is collected by the accelerometer, and the sampling frequency is 12 kHz. The bearings selected in this paper are installed at the drive end, and there are three types of bearings: inner ring fault bearing, outer ring fault bearing and normal bearing. The parameters are shown in Table 2.

**Figure 6:**
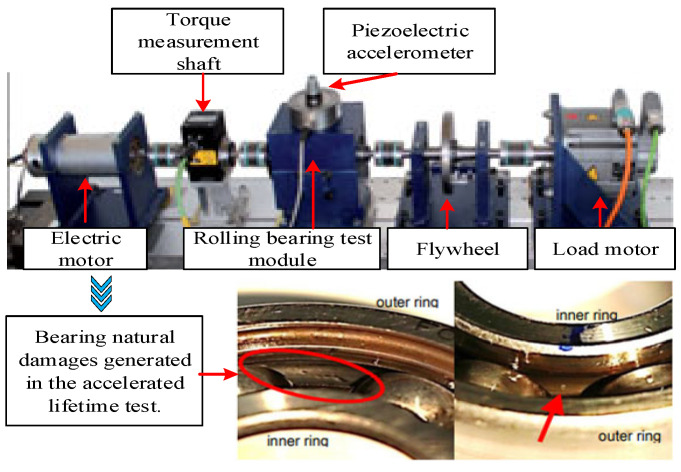
Modular test bench of PU.

On the modular test bench (Figure 6), the Paderborn University (PU) researchers with 6 sets of normal bearing data, 12 sets of artificially damaged bearing data of three fault types, and 14 groups of naturally damaged bearing data caused by accelerating lifetime test [35]. Damage levels are divided according to the percentage of length of the damage relative to pitch circumference is calculated (Table 3). The vibration acceleration signal of the faulty bearing is collected by the accelerometer, and the sampling frequency is 64 kHz. We choose the natural damage dataset of PU as the target domain data, and the details of the parameters are shown in Table 4.

**Figure 7:**
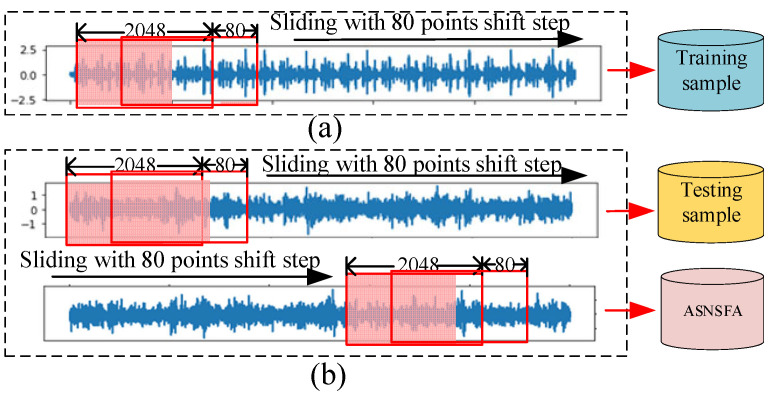
Data processing: (**a**) source domain data processing, (**b**) target domain data processing.

We sampled and processed the CWRU source domain data in Table 2, taking all 2048 data points as a sample. Because there are not enough data points in the original data, the number of samples that can be intercepted is too small, and when the number of training samples is very small, it is easy to cause over-fitting. Therefore, we use the method of overlapping sampling as shown in Figure 7a. There is a partial overlap between each sample and the subsequent sample, with an offset of 80 and the training samples are obtained. Similarly, we process the PU natural damage fault data as shown in Figure 7b. Finally, the testing samples are obtained and a small number of samples for adjustment support set (SNSASS) are obtained. It is worth noting that the testing samples and SNSASS are independent and not duplicated. SNSASS can be seen as a small number of samples of real machines that can be obtained. The experimental samples are shown in Table 5.

### 4.2. S(s), S(s+t) and S(t) Analysis

To verify the validity of our proposed transfer method, we performed the following three experiments as shown in Table 6.

(1) S(s): direct transfer method (baseline).

Direct transfer method is a simple method without any optimization and adjustment of fixed network parameters. This method uses source domain data for training and directly uses target domain data for testing. In this experiment, the support set of the direct transfer method based on a Siamese network is shown Equation (12), and the samples are all training samples from the source domain. Direct transfer method based on the Siamese network is expressed by S(s).

(12) $$S=\left\{\left(x_{s1},y_{s1}\right),\left(x_{s2},y_{s2}\right)\ldots ,\left(x_{sk},y_{sk}\right)\right\}$$

The *s* in $\;x_{s}$ represents from the source domain.

In the experiment of S(s), we use the ASF data from CWRU to train and learn in the Adam optimization program, the epochs of training are 90, and batch size chooses 64, and the diagnostic knowledge learned is fixed. In the testing process, we directly input the NF samples provided by PU into the model for feature extraction, and then select the samples that are most similar to the test samples from the support set (the samples in the support set are training samples), and think that they are the same class.

(2) S(s+t): directly add target domain samples to the support set.

The training process is the same as that of (1). Before testing, however, SNSASS are added to the support set as a classification reference. The testing process is the same as that of (1), except that the support set contains both training samples and SNSASS.

(3) S(t): replace the original samples in the support set with the target domain samples.

In the experiment of S(t), the process of training stage is consistent with that of (1), and then all the samples of the original support set are replaced by SNSASS (the sample in the support set at this time is SNSASS). In the process of testing, input PU samples to test and obtain the results.

**Figure 8:**
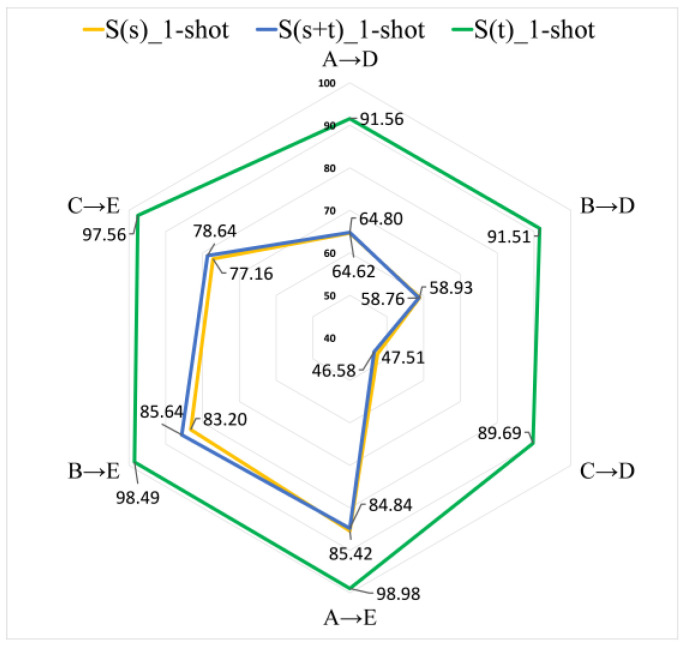
The results of S(s) (baseline), S(s+t) and S(t) in different transfer learning tasks.

First of all, we verify the performance of S(s) (baseline), S(s+t) and S(t) in A→D, B→D, C→D, A→E, B→E and C→E transfer tasks, each experiment is carried out 10 times, and finally take the average. The experimental results are shown in Figure 8.

It can be seen from Figure 8 that S(t) has an absolute advantage in all tasks. The accuracy is more than 89.69%, which is much higher than the other two methods, 42.18% higher than S(s) in C→D. This is because, based on the S(t) learning theory, the instances of the support set are all SNSASS $\left(x_{t},y_{t}\right)$ from the target domain, and the spatial distribution of the feature space of the test samples $\hat{x}$ that need to be classified is very similar to that of $x_{t}$, so it is easy to find similar examples in the support set and regard them as the same class of fault. The experimental results of S(s) and S(s+t) are very close, but in most cases the accuracy of S(s+t) is slightly higher than that of S(s). This is because the support set of S(s+t) has a small number of SNSASS. Based on the few-shot learning theory (see Section 2), these SNSASS can help the test sample $\;\hat{x}$ to better find the most similar class to itself. However, its number accounts for a small proportion (see Equation (13), $\eta =\frac{15}{1980+15}\approx 0.75\%$) in the support set, which cannot bring great performance improvement as S(t) ($\eta =\frac{15}{15}=100\%$) does.

(13) $$\eta =\frac{n_{SNSASS}}{n_{SNSASS}+n_{Training}}$$

where η is the proportion of the number of SNSASS in the total quantity. $n_{SNSASS}$ is the number of SNSASS. $n_{Training}$ is the number of training sample.

**Figure 9:**
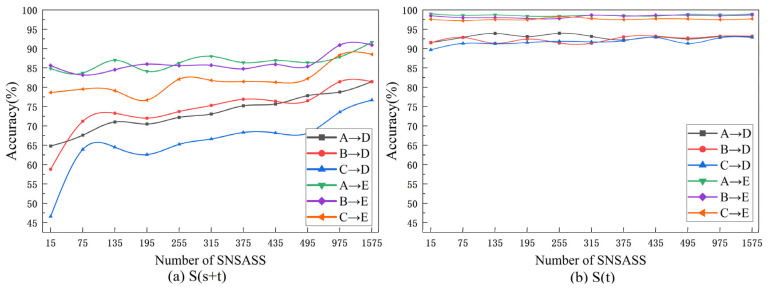
The curve of accuracy with the increase in the number of SNSASS. (**a**) the results of S(s+t). (**b**) the results of S(t).

In order to further verify the effect of η on S(s+t) and S(t), we gradually increase the number of SNSASS and repeat the experiment again, each experiment is repeated 10 times, and the result is shown in Figure 9. As can be seen from Figure 9a, with the increase in the number of SNSASS (η increase), the accuracy of S(s+t) does not increase linearly, but it shows an increasing trend as a whole, especially in A→D, B→D and C→D experiments. However, with the increase in SNSASS, the performance of S(t) has not been improved as shown in Figure 9b, and the accuracy fluctuates within an allowable error range. In other words, if we can obtain a small amount of target domain data, S(t) can give full play to its performance.

### 4.3. Comparisons with Other Methods

**Figure 10:**
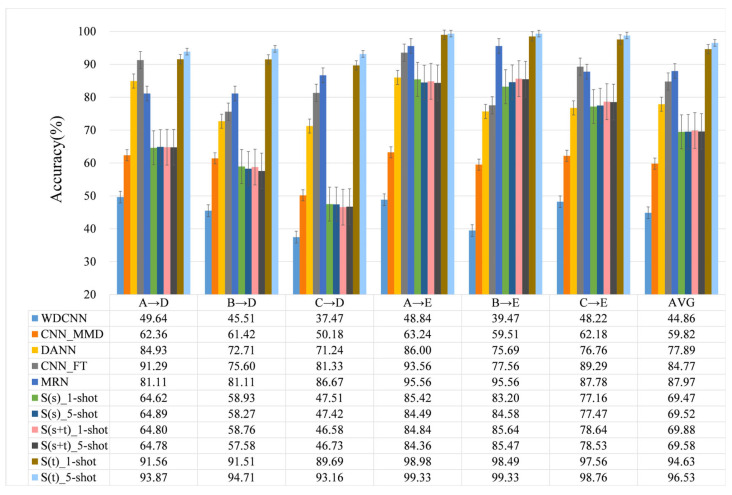
Experimental results.

We also contrast our method with some popular methods, which include WDCNN [18,33], CNN_MMD [36], CNN_FT [37], DANN [38] and MRN [30]. It should be noted that we set the experimental parameters to the best case according to the characteristics of each method, including data format, hyperparameters, epochs, and so on. The number of training samples is 1980, the number of SNSASS is 15, and the number of test samples is 225. Similarly, each method is tested 10 times in the A→D, B→D, C→D, A→E, B→E and C→E transfer tasks in turn, and the results are averaged. The experimental results are shown in Figure 10.

Experiments show that S(t)_5-shot achieves the highest accuracy in all transfer learning tasks, with an average of 96.53%, and S(t)_1-shot ranks second with an average of 94.63%, followed by MRU, CNN_FT, DANN, S(s+t), S(s), CNN_MMD. There is no doubt that WDCNN performs the worst among all transfer learning tasks, with an average accuracy of only 44.86%. Of course, we know that it is unfair to compare WDCNN with these advanced methods, but it also reflects the difficulty of these transfer learning tasks. After all, there is a big gap between the fault features of ASF and those of NF. It is also evident from the figure that in almost all methods (except WDCNN) the results of A→D, B→D and C→D are worse than A→E, B→E and C→E, the reason is that D’s lower damage (level 1 damage) level than E (level 2 damage), E’s more serious damage and more obvious failure features. Learning the knowledge of A, B and C breakdown to diagnose E would be better.

**Figure 11:**
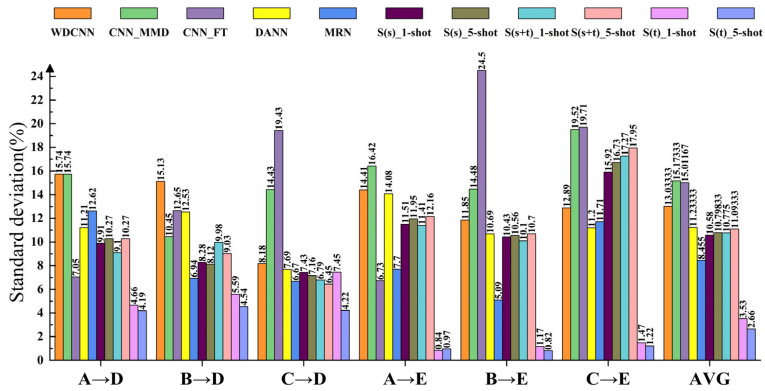
The standard deviation of 10 repeated experiments for each method.

Figure 11 shows the standard deviation of 10 repeated experiments for each method in the A→D, B→D, C→D, A→E, B→E and C→E transfer learning tasks. As can be seen from Figure 11, S(t)_5-shot has the smallest standard deviation among all transfer learning tasks with an average of 2.66%, followed by S(t)_1-shot with an average of 3.53%, much smaller than other methods. Except that the average standard deviation of MRN is 8.45%, the rest are more than 10%, which means that it is difficult to learn diagnosis knowledge from ASF to diagnose NF, resulting in very unstable diagnosis results of other methods. Simultaneously, it is demonstrated that S(t) has much higher stability than other methods.

### 4.4. The Influence of Different Source Domain and the Number of Training Samples

**Figure 12:**
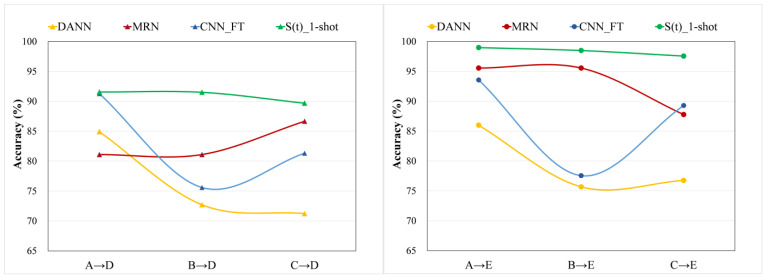
The variation of experimental results with different source domains.

It is not only the quality of the data in the source domain that is very important, but the quantity of the data is also important. It is very important to select the appropriate bearing fault source domain data and quantitative training model, but in the actual industrial production, it is difficult to determine the appropriate source domain data. Therefore, in this section we discuss the harshness of the proposed method on the source domain data. We selected several relatively well-performing methods for comparison, Figure 12 is the result curve of DANN, MRN, CNN_FT and S(t)_1-shot learning fault diagnosis knowledge from datasets A, B and C, respectively, and used it to diagnose D and E. It can be clearly seen that DANN, MRN and CNN_FT learns knowledge from different source domains and fixes the model, which leads to great differences in experimental results. The reason is that there is a big gap between the working conditions of A (1772 rpm), B (1750 rpm) and C (1730 rpm) in speed. However, compared with other methods, the result of S(t)_1-shot learning from A, B and C to diagnose D and E has only a small change and a slight downward trend, indicating that S(t) has good ability to learn and can be well leverage knowledge of the source domain. The reasons for the slight downward trend with A-B-C are as follows: according to the speed of A, B and C, we think that the working condition of A is more complex than that of B, and that of B is more complex than that of C. The model can learn more obvious fault features under more complex working conditions, so as to better complete the transfer task. In [18,21,39], the authors have also obtained a similar conclusion.

**Figure 13:**
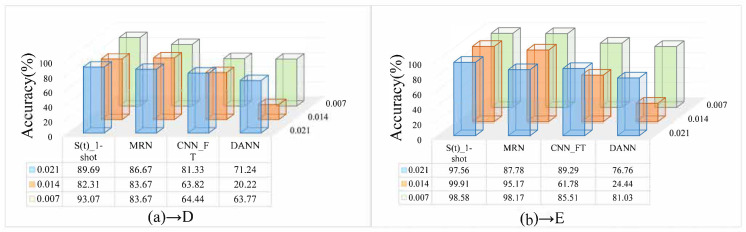
Results of training in different fault degrees, (**a**) tested in D. (**b**) tested in E.

More complex working condition will have more diagnostic knowledge. Next, we want to explore the influence of different fault diameters of the source domain bearing. Therefore, an additional small experiment was performed here. Following the principle of control variables, CWRU data (the load is 3 hp) with fault diameters of 0.021, 0.014, and 0.007 inches were used as training sets and tested in D and E, the result is shown in Figure 13.

As can be seen in Figure 13, S(t) effectively learns diagnostic knowledge from different fault diameters and shows the best performance, followed by MRN, and the worst is DANN, which surprises us with just over 20% in 0.014 inches. However, we failed to find the rule that the fault diameters affect the performance of the model, which may be due to the big difference between ASF and NF.

**Figure 14:**
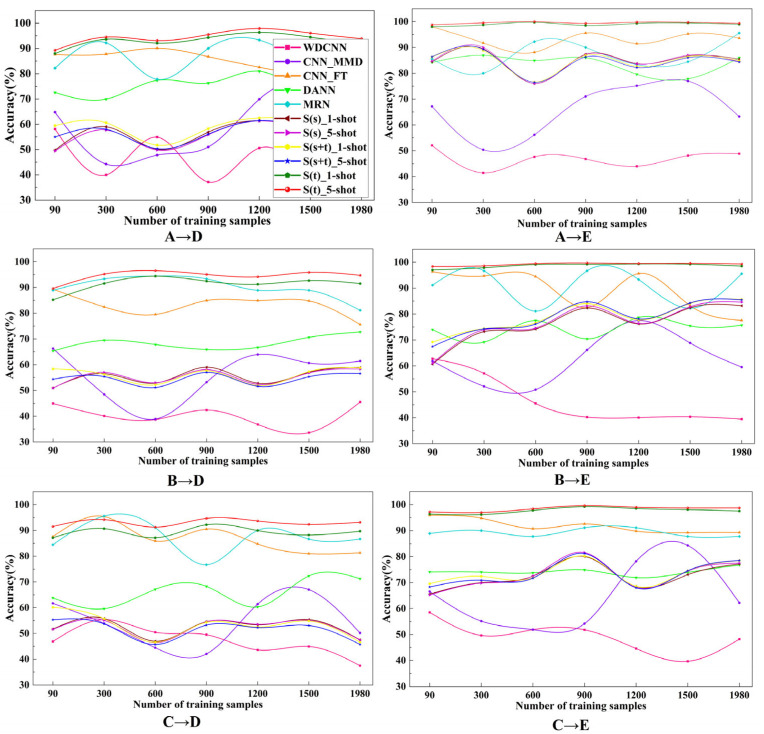
Variation of diagnostic results with different numbers of training samples.

In order to explore the performance of various methods under different sample numbers, the following groups of experiments were carried out when the number of training samples was 90, 300, 600, 1200, 1500 and 1980. As shown in Figure 14, it presents the curve of all experimental results with the number of training samples. Incredibly, the experimental results do not improve with the increase in the number of training samples, but show a special curve shape. It is because having too small a number of training samples will cause the model to learn insufficient knowledge that can be used in the target domain, resulting in poor performance when diagnosing in the target domain; and having too many training samples will cause the learned knowledge to be too focused on the source domain, which is not applicable when it is transferred to the target domain. However, compared with other methods, the results of S(t) do not change greatly with the number of data samples, which shows that the dependence of S(t) method on data samples is very small and stable. This is because the few-shot learning strategy of S(t) can learn and use knowledge in a small number of training samples and is not sensitive to the growth of data.

Assuming that a small sample of the target domain is obtained, similarly to fine-tuning-based methods, S(t) can improve the performance of the model in transfer learning. However, after obtaining the new target domain data, fine-tuning-based methods still need to train the models that have been trained in the source domain. The S(t) method does not need a second training, but only needs to input these small target domain samples into the support set, which is more convenient than fine-tuning-based methods in some practical applications.

## 5. Conclusions

In this paper, it is established that there is still a long distance between the research of intelligent fault diagnosis and the practical industrial application. A bearing fault diagnosis based on few-shot transfer learning across different datasets is proposed, which uses a very small number of target domain samples to adjust the support set to improve the generalization performance of the model. Many groups of transfer experiments are carried out by using the ASF dataset of CWRU and the NF dataset of PU. The conclusions are as follows:

(1) With only a small amount of SNSASS, S(t) method greatly improves the accuracy of fault classification, and the accuracy of S(s+t) is not significantly improved, but increases with the increase in the number of SNSASS.
(2) Compared with other methods, the proposed S(t) method has the highest accuracy in all cases and is also the most stable method.
(3) S(t) can fully learn diagnostic knowledge in different source domains and sample numbers, and effectively use this knowledge to identify the health state of the target bearing, which has strong generalization and robustness. In addition, unlike the fine-tuning-based method, S(t) does not need secondary training, which is more convenient in some practical applications.

S(t) provides a feasible way to apply laboratory data knowledge to real machine fault diagnosis, solves the difficulty that a large amount of data cannot be collected in the real world, and also provides a new idea and method for transfer learning. However, obtaining a small amount of target domain data (SNSASS) is the key to the S(t) method. In some cases of actual industrial production, it is also not easy to obtain a small amount of target domain data, which is a limitation of the S(t) method. At the same time, although the difference between ASF and NF brings great challenges to the transfer learning tasks, because of the lack of available data, we were only able to perform three classification tasks. More classification experiments and verification in more datasets can be performed in the future.

## Figures and Tables

**Table 1:** Structural parameter of WDCNN.

No	Layer Type	Kernel Size/Stride	Kernel Number	Output Size (Width × Depth)	Padding
1	Conv1	64 × 1/16 × 1	16	128 × 16	same
2	Pooling1	2 × 1/2 × 1	16	64 × 16	valid
3	Conv2	3 × 1/1 × 1	32	64 × 32	same
4	Pooling2	2 × 1/2 × 1	32	32 × 32	valid
5	Conv3	3 × 1/1 × 1	64	32 × 64	same
6	Pooling3	2 × 1/2 × 1	64	16 × 64	valid
7	Conv4	3 × 1/1 × 1	64	16 × 64	same
8	Pooling4	2 × 1/2 × 1	64	8 × 64	valid
9	Conv5	3 × 1/1 × 1	64	6 × 64	valid
10	Pooling5	2 × 1/2 × 1	64	3 × 64	valid
11	Fully-connected	100	1	100 × 1	

**Table 2:** Source domain data parameters.

Dataset Name	Name	Fault Location	Speed (rpm)	Loads (hp)
	0.021-OuterRace	Outer ring	1772	1
A	0.021-InnerRace	Inner ring	1772	1
	Normal	None	1772	1
	0.021-OuterRace	Outer ring	1750	2
B	0.021-InnerRace	Inner ring	1750	2
	Normal	None	1750	2
	0.021-OuterRace	Outer ring	1730	3
C	0.021-InnerRace	Inner ring	1730	3
	Normal	None	1730	3

**Table 3:** Damage levels to determine the extent of damage.

Damage Level	Assigned Percentage Values	Limits for Bearing 6203
1	0–2%	≤2 mm
2	2–5%	>2 mm

**Table 4:** Target domain data parameters.

Dataset Name	Name	Fault Location	Damage (Main Mode and Symptom)	Damage Level	Damage Feature	Load Torque (Nm)	Speed (rpm)	Radial Force (N)
D	KI04	Inner ring	Fatigue: pitting	1	Single	0.7	1500	1000
	KA04	Outer ring	Fatigue: pitting	1	Single	0.7	1500	1000
	K005	Normal	None	None	None	0.7	1500	1000
E	KI16	Inner ring	Fatigue: pitting	2	Single	0.7	1500	1000
	KA16	Outer ring	Fatigue: pitting	2	Single	0.7	1500	1000
	K004	Normal	None	None	None	0.7	1500	1000

**Table 5:** Experimental samples.

	Sample Purpose	Inner Ring 0	Outer Ring 1	Normal 2	Total
Source domain	Training	660	660	660	1980
Target domain	SNSASS	5	5	5	15
	Testing	75	75	75	225

**Table 6:** Eight kinds of experiments.

Number	Experiment Name	Model	Support Set
1	S(s)	Siamese network	Training sample
2	S(s+t)	Siamese network	Training sample and SNSASS
3	S(t)	Siamese network	SNSASS
